# What is the optimal radiation dose for non-operable esophageal cancer? Dissecting the evidence in a meta-analysis

**DOI:** 10.18632/oncotarget.18760

**Published:** 2017-06-28

**Authors:** Yong Chen, Hui-Ping Zhu, Tao Wang, Chang-Jiang Sun, Xiao-Lin Ge, Ling-Feng Min, Xian-Wen Zhang, Qing-Qing Jia, Jie Yu, Jian-Qi Yang, Heike Allgayer, Mohammed L. Abba, Xi-Zhi Zhang, Xin-Chen Sun

**Affiliations:** ^1^ Department of Medical Oncology, Northern Jiangsu People's Hospital, Clinical Medical College of Yangzhou University, Yangzhou, Jiangsu, China; ^2^ Department of Radiotherapy, The First Affiliated Hospital of Nanjing Medical University, Nanjing, Jiangsu, China; ^3^ Department of Medical Oncology, Zhangjiagang First People's Hospital, Zhangjiagang, Jiangsu, China; ^4^ Department of Respiratory Medicine, Northern Jiangsu People's Hospital, Clinical Medical College of Yangzhou University, Yangzhou, Jiangsu, China; ^5^ Department of Experimental Surgery-Cancer Metastasis, Medical Faculty Mannheim, University of Heidelberg, Germany and Centre for Biomedicine and Medical Technology, Medical Faculty Mannheim, University of Heidelberg, Germany

**Keywords:** esophageal cancer, radiation dose, meta-analysis, chemoradiotherapy, survival benefit

## Abstract

The standard radiation dose 50.4 Gy with concurrent chemotherapy for localized inoperable esophageal cancer as supported by INT-0123 trail is now being challenged since a radiation dose above 50 Gy has been successfully administered with an observable dose–response relationship and insignificant untoward effects. Therefore, to ascertain the treatment benefits of different radiation doses, we performed a meta-analysis with 18 relative publications. According to our findings, a dose between 50 and 70 Gy appears optimal and patients who received ≥ 60 Gy radiation had a significantly better prognosis (pooled HR = 0.78, *P* = 0.004) as compared with < 60 Gy, especially in Asian countries (pooled HR = 0.75, *P* = 0.003). However, contradictory results of treatment benefit for ≥ 60 Gy were observed in two studies from Western countries, and the pooled treatment benefit of ≥ 60 Gy radiation was inconclusive (pooled HR = 0.86, *P* = 0.64). There was a marginal benefit in locoregional control in those treated with high dose (> 50.4/51 Gy) radiation when compared with those treated with low dose (≤ 50.4/51 Gy) radiation (pooled OR = 0.71, *P* = 0.06). Patients that received ≥ 60 Gy radiation had better locoregional control (OR = 0.29, *P* = 0.001), and for distant metastasis control, neither the > 50.4 Gy nor the ≥ 60 Gy treated group had any treatment benefit as compared to the groups that received ≤ 50.4 Gy and < 60 Gy group respectively. Taken together, a dose range of 50 to 70 Gy radiation with CCRT is recommended for non-operable EC patients. A dose of ≥ 60 Gy appears to be better in improving overall survival and locoregional control, especially in Asian countries, while the benefit of ≥ 60 Gy radiation in Western countries still remains controversial.

## INTRODUCTION

Esophageal cancer (EC) has a very high incidence and mortality rate worldwide. Approximately 75% of all cases occur in Asia with China bearing the largest burden, accounting for about 50% of the total cases and cancer specific deaths [1]. Surgery still remains the main curative treatment modality; however, nearly 66% of newly diagnosed patients have regional or distant metastasis at presentation [2]. The relative 5-year overall survival rate has been hovering at around 19% in the USA since the year 2000 [2].

For patients with localized and locally advanced inoperable disease, the worldwide consensus standard treatment recommendation, as sturdily supported by the RTOG 85–01 trial, is definitive concurrent chemoradiotherapy (CCRT) [3]. The optimal radiation dose for locally advanced EC was subsequently explored by the INT-0123 randomized controlled trial (RCT) (also known as RTOG 94-05) [4] and their analysis showed no benefit in the high dose (HD) treatment arm (64.8 Gy) as compared to the standard dose of 50.4 Gy. Since then, a dose of 50.4 Gy has been recommended as the standard dose for locally advanced EC in the American guidelines as “evidence-based”. Additional data supporting this dose is lacking despite debates about its clinical validity [5]. Meanwhile, the National Comprehensive Cancer Network advocates this same dose for preoperative radiotherapy (available from: https://www.nccn.org). Up until the time of putting together this meta-analysis, there were no RCTs that looked at evaluating varying radiation doses in non-surgical patients [6, 7]. A recent systematic review evaluated 27 studies comprising a total of 1972 patients who received ≥ 60 Gy CCRT and found that ≥ 60 Gy CCRT improved clinical outcomes as compared to the 50∼54 Gy CCRT arm [8]. Since several studies have successfully administered a radiation dose above 50 Gy without significant untoward effects, and a dose–response relationship has been observed with increasing doses above 50 Gy, a further dose escalation might be justifiable on its potential merits [7].

In this study, we present a meta-analysis evaluating the clinical benefits of different radiation doses in a large group of patients globally, and use the findings to project, and articulate what might be considered as the optimal radiation dose for non-surgical EC.

## RESULTS

### Search results and description of studies

**Figure 1:**
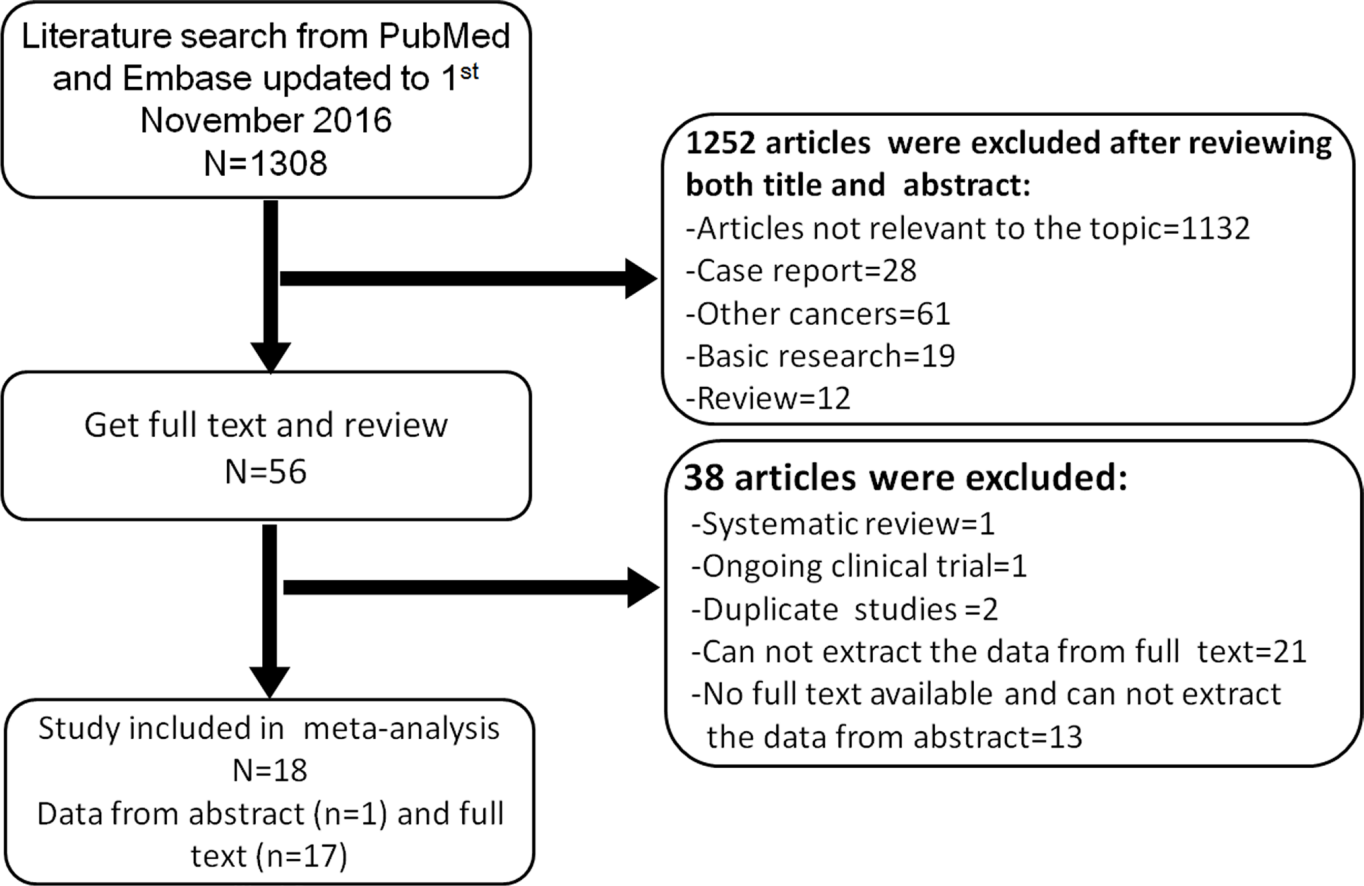
Literature search strategy and study selection for the meta-analysis

We identified 1308 potentially relevant articles from the database. Eventually, 18 articles were selected in the final analysis by the review group after examination of the titles, abstracts and full-texts. 1290 articles were excluded for the reasons stated in Figure 1. An overview of all the included studies is shown in Table 1. All of the studies were published from 1998 to 2016, and comprised 1 population based propensity-score matched analysis, 1 RCT (INT-0123) and 16 retrospective studies. There were 9 studies from Asian countries (4 from Chinese region, 2 studies from Japan, 1 from South Korea, 1 from Turkey and 1 from Iran), and 9 from Western countries (5 studies from USA, 2 from France, 1 from Canada and 1 from Germany). A total of 2846 EC patients were included in these studies. The total delivered radiation dose ranged from 8.5 to 100.8 Gy. Five studies reported the occurrence (frequency) of locoregional failure (LRF) and 4 studies reported that of distant metastasis failure (DMF) in high dose (HD) and low dose (LD) groups. The hazard ratios (HRs) and their corresponding 95% confidence intervals (CIs) for overall survival (OS) could be directly or indirectly obtained from all the 18 studies. However, in the INT-0123 trial, the HR and its 95% CI were estimated based on the Kaplan-Meier curves of patients receiving the assigned dose of radiation. The thresholds between HD and LD from each study were mainly around 50 Gy or 60 Gy. In Asian studies, there were 6 studies analyzing the survival benefit of radiation dose ≥ 60 Gy compared with < 60 Gy. Additionally, there were 3 studies that compared the survival benefit of 50 Gy as a threshold of radiation dose. In contrast, there were 6 studies comparing the survival benefit of radiation dose higher than circa 50 Gy with lower than circa 50 Gy in Western countries, 2 studies analyzing the survival benefit of radiation dose ≥ 60 Gy, and only 1 study analyzing the survival benefit of 70 Gy.

**Table 1:** Overview of the studies included in the meta-analysis

Author	Year	Sample size (LD/HD)	Median Age	Study period	Geographic area	Radiation technology	Median dose (Gy)	Chemotherapy regimens	Radiation dose (LD/HD, Gy)	Pathological types	Clinical stage	Median follow-up (month)	Quality*	Reference
Tanisada K (<75Y-cohort)	1998	37/125	64	1992-1994	Japan	-	60	44%with CT	<60/≥60	SCC/Ad/Ad-SCC	Ⅰ-Ⅲ	0.46^@^	7	[9]
Tanisada K (≥75Y-cohort)	1998	27/63	80	1992-1994	Japan	-	60	22%with CT	<60/≥60	SCC/Ad/Ad-SCC	Ⅰ-Ⅲ	0.46^@^	7	[9]
Minsky BD	2002	109/109	-	1995-1999	USA	Multiple field technique	-	5-FU/CDDP	50.4/64.8	SCC/Ad	T1-4N0-1M0	16.4	5^#^	[4]
Zhang Z	2005	43/26	-	1990-1998	USA	-	-	5-FU based or other	30-51.0/54.0-64.8	SCC/Ad/other	Ⅱ-Ⅲ	22	7	[10]
Nallapareddy	2005	11/19	72.5	1999-2004	USA	-	-	Paclitaxel alone or 5-FU alone or with CDDP/oxaliplatin	<50.4/≥50.4	SCC/Ad	Ⅰ-Ⅳ	10	6	[11]
Wang S	2006	11/24	64	1985-2001	USA	Conventional techniques/3D	-	5-FU or paclitaxel based	<50/≥50	SCC/Ad	Ⅰ-Ⅲ	39	6	[12]
Di Fiore F	2007	105^&^	-	1997-2003	France	-	43.5	5-FU /Irinotecan +CDDP	≤50/>50	SCC/Ad	Ⅰ-Ⅲ	-	8	[13]
Huang SH	2008	21/29	68	1997-2005	Canada	Hypofractionated RT /3D/IMRT	-	5-FU-based or CDDP-based	54/70	SCC/Ad	Ⅰ-Ⅳ	3.3^@^	6	[14]
Shen WB	2011	20/48	60	2003-2008	China	2D/3D/IMRT	60	51.5% CT with CDDP based	<60/≥60	SCC	with supraclavicular LNM	15	6	[15]
Semrau R	2012	203^&^	63	1995-2005	Germany	2D/3D	-	57.1% CT with 5-FU/CDDP	40–59.9/≥60	SCC/Ad	Ⅰ-Ⅳ	47.9	7	[16]
Mirinezhad	2013	151/111	-	2006-2011	Iran	-	44	65.5% CT with CDDP or 5-FU or combined or other	<50/≥50	SCC/Ad	T2-3N0-1	-	7	[17]
Clavier JB	2013	60/83	-	2003-2006	France	3D	-	CDDP/5FU/Taxane	38-50.4/50.7-72	SCC/Ad	Ⅰ-ⅣA	20.8	7	[18]
He L	2014	137/56	68	1998-2012	USA	3D/IMRT /proton beam	50.4	5-FU with platin/taxane	41.4-50.4/52.2-66	SCC/Ad	Ⅰ-Ⅳ	32.4	8	[19]
Suh YG	2014	49/77	-	1998-2008	South Korea	2D/3D	-	5-FU based or other	45-59.4/60-75.6	SCC/Ad/unknown	Ⅱ-Ⅲ	-	7	[20]
Xu H	2014	16/21	76	2003-2012	China	2D/3D/IMRT /IGRT/VMAT	51.5	54.1% CT with 5-FU or paclitaxel based	≤50/>50	SCC	Ⅰ-Ⅳ	64^$^	5	[21]
Li X	2015	40/76	76	2008-2013	China	3D/IMRT	60	5-FU or taxane based	<60/≥60	SCC	I-Ⅳ	16.97	7	[22]
Chen CY	2016	324/324	-	2008-2013	Taiwan	3D/IMRT/IGRT	-	-	50–50.4/≥60	SCC	Ⅱ-ⅣA	-	8	[23]
Gemici C	2016	38/11	-	-	Turkey	2D/3D	-	CDDP based or paclitaxel+5-FU	40-50/50.01-60	SCC/Ad	T3-4N0-1	-	6	[24]
Hirano H	2016	62/180	-	2000-2011	Japan	-	-	CDDP with 5-FU	50.4/64.8	SCC	Ⅱ-Ⅲ	-	8	[25]

LD: low dose; HD: high dose; 2D: two dimensional radiation therapy; 3D: three dimensional conformal radiotherapy; IMRT: intensity-modulated radiation therapy; IGRT: image guided radiation therapy; VMAT: volumetric modulated Arc therapy; CT: chemotherapy; CDDP: cisplatin; 5-FU: 5-Fluorouracil; EC: esophageal cancer; SCC: squamous cell carcinomas; Ad: adenocarcinoma; LNM: lymph node metastasis; & in total; @ Year; $ week.

*The quality of non-radomized studies were evaluated by the 9-star Newcastle-Ottawa Scale. # Quality was assessed by the JADAD scale.

### Quality assessment

The quality scores of included studies are summarized in Table 1. The quality of the randomized controlled study (INT-0123 trial) was high, scoring 5 according to the JADAD scale, while the quality scores of the 17 non-randomized studies ranged from 5 to 8, with a median score of 7. Altogether, all the 18 studies had medium to high quality.

### Association of radiation dose with OS

**Figure 2:**
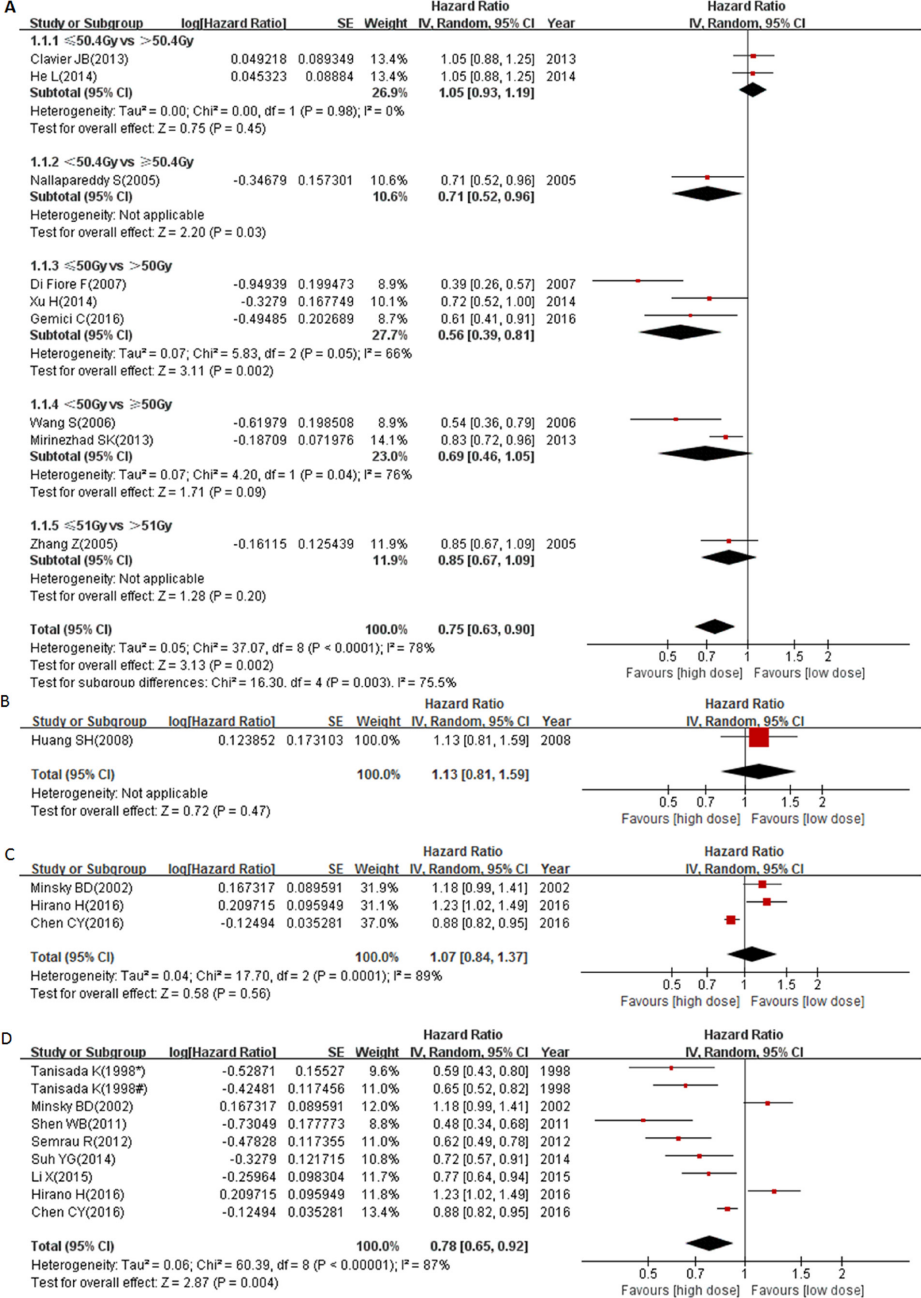
Forest plot describing the association between OS and (< circa 50 Gy vs ≥ circa 50 Gy) subgroup (**A**), (54 Gy vs 70 Gy) subgroup (**B**), (50–50.4 Gy vs ≥ 60 Gy) subgroup (**C**) and (< 60 Gy vs ≥ 60 Gy) subgroup (**D**). * ≥ 75 years cohort; # < 75 years cohort.

Different cut-offs were used in grouping high and low radiation dose patients in the 18 selected studies. Therefore, we divided the 18 studies into 4 groups based on the threshold of grouping (< circa 50 Gy vs ≥ circa 50 Gy; 54 Gy vs 70 Gy; 50–50.4 Gy vs ≥ 60 Gy and < 60 Gy vs ≥ 60 Gy). As shown in Figure 2A, the patients who received ≥ circa 50 Gy radiation survived better (pooled HR = 0.75, 95% CI: 0.63–0.90, *P* = 0.002). Furthermore, patients that received 70 Gy radiation had no overall survival benefit when compared with patients that received 54 Gy radiation (HR = 1.13, 95% CI: 0.81–1.59, *P* = 0.47, Figure 2B). These results indicated that 50–70 Gy radiation appeared to be suitable for curative purpose in non-operable EC patients. Since 50.4 Gy is currently considered as a standard dose for EC treatment according to the INT-0123 trial, we further analyzed the survival benefit of ≥ 60 Gy group as compared to the 50–50.4 Gy group. Our results showed that patients that received ≥ 60 Gy radiation had no overall survival benefit when compared with those in the 50–50.4 Gy group (pooled HR = 1.07, 95% CI: 0.84–1.37, *P* = 0.56, Figure 2C). Considering that there were other studies exploring the survival benefit of ≥ 60 Gy group compared with < 60 Gy group, we combined the studies of 50–50.4 Gy vs ≥ 60 Gy with the studies of < 60 Gy vs ≥ 60 Gy together to analyze the potential survival benefit of ≥ 60 Gy radiation in EC patients. Altogether, there were 8 studies comparing the survival of < 60 Gy group with ≥ 60 Gy group including those studies that specifically compared 50–50.4 Gy and ≥ 60 Gy radiation doses. Our results revealed that patients who received ≥ 60 Gy radiation had significantly better prognosis (pooled HR = 0.78, 95% CI: 0.65–0.92, *P* = 0.004, Figure 2D). However, our results showed that there was significant heterogeneity in these meta-analyses (50–50.4 Gy vs ≥ 60 Gy: *P* = 0.0001, I^2^ = 89%; < 60 Gy vs ≥ 60 Gy: *P* < 0.00001, I^2^ = 87%). Meanwhile, there was also an unbalanced geographic area distribution in the above subgroup meta-analysis. In the 50∼50.4 Gy vs ≥ 60 Gy subgroup, there was only one study from a Western country, while with the exception of one, all studies in the < 60 Gy vs ≥ 60 Gy subgroup were from Asian countries. So we conducted a subgroup meta-analysis to explore whether the heterogeneity was due to differences of geographic area.

**Figure 3:**
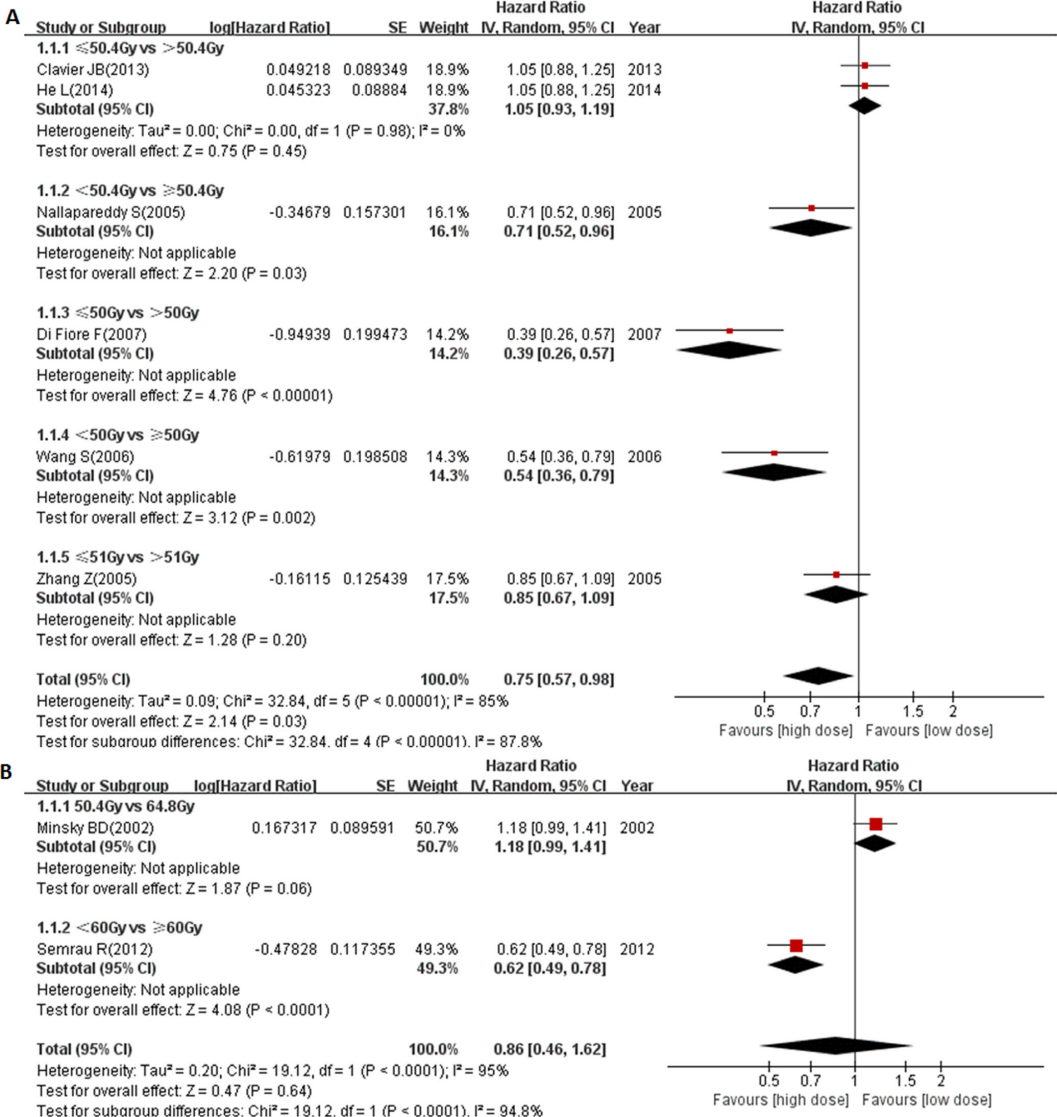
Forest plot describing the association between OS and (< circa 50 Gy vs ≥ circa 50 Gy) subgroup (**A**) and (< 60 Gy vs ≥ 60 Gy) subgroup (**B**) from Western countries.

Overall, there were 9 studies from Western countries. As shown in Figure 3A, patients received ≥ circa 50 Gy radiation had significantly better outcomes (pooled HR = 0.75, 95% CI: 0.57–0.98, *P* = 0.03), and patients who received 70 Gy radiation had no survival benefit (Figure 2B). Our results also demonstrated a marginally significant survival benefit in the 50.4 Gy group compared to the 64.8 Gy group (HR = 1.18, 95% CI: 0.99–1.41, *P* = 0.06, Figure 3B). In contrast, another study showed that there was a significant survival benefit in ≥ 60 Gy group when compared with < 60 Gy group (HR = 0.62, 95% CI: 0.49–0.78, *P*< 0.0001, Figure 3B). Altogether, we combined the study of 50.4 Gy vs 64.8 Gy with study of < 60 Gy vs ≥ 60 Gy together to analyze the pooled survival benefit of ≥ 60 Gy radiation. As shown in Figure 3B, there was no significant benefit of ≥ 60 Gy radiation in Western countries (pooled HR = 0.86, 95% CI: 0.46–1.62, *P* = 0.64). These results suggest that the optimal radiation dose in Western EC patients still needs further investigation. However, it was worth noting that a more advanced radiation technology platform (three-dimensional planning radiation) was used from 2003 instead of anterior-posterior field in Semrau’s study [16].

**Figure 4:**
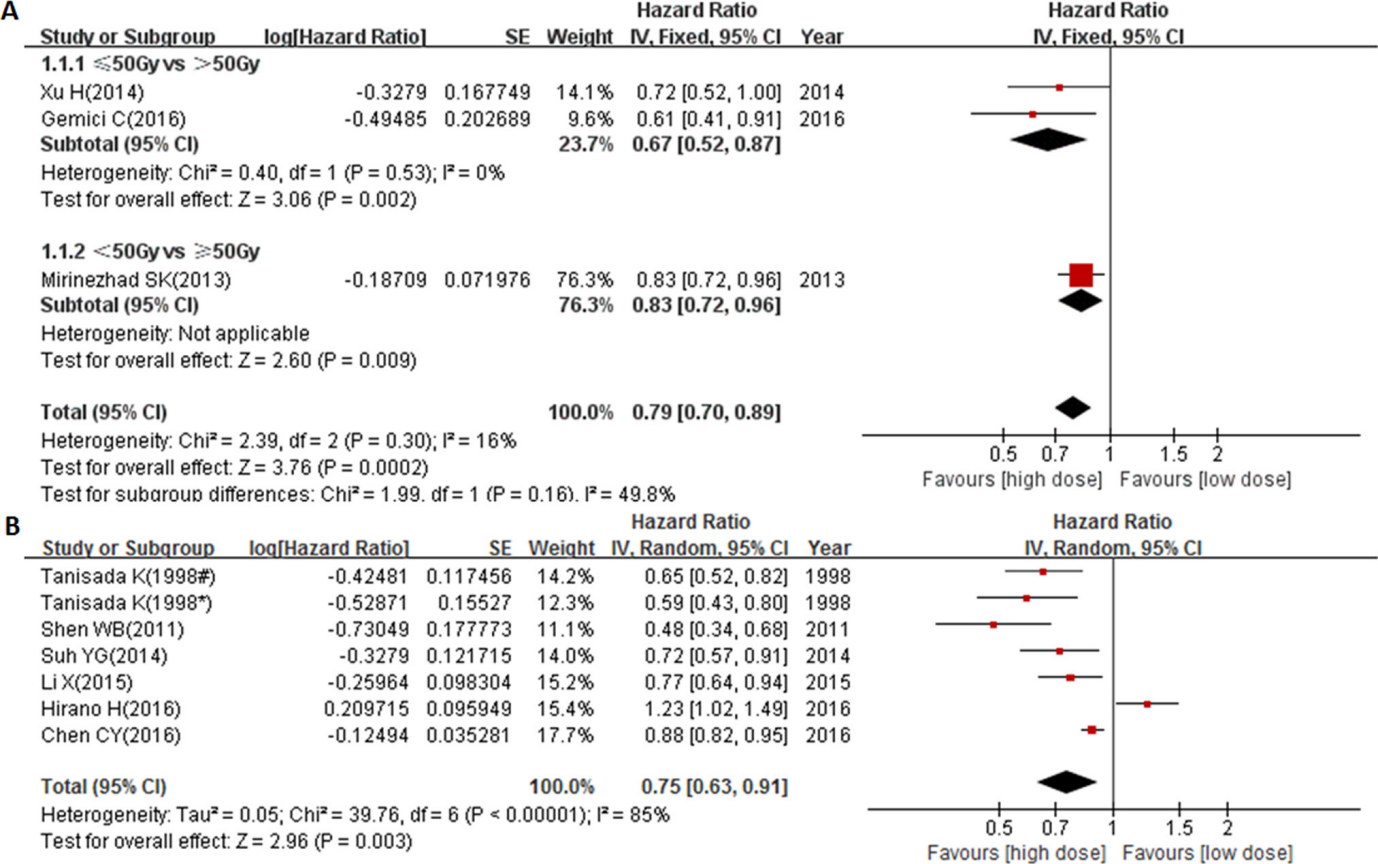
Forest plot describing the association between OS and (< circa 50 Gy vs ≥ circa 50 Gy) subgroup (**A**) and (< 60 Gy vs ≥ 60 Gy) subgroup (**B**) from Asian countries. * ≥ 75 years cohort; # < 75 years cohort.

There were also 9 studies from Asian countries. As shown in Figure 4A, similar results suggesting that the optimal radiation dose should not be lower than 50 Gy were also obtained in the Asian countries (pooled HR = 0.79, 95% CI: 0.70–0.89, *P* = 0.0002). Moreover, patients who received ≥ 60 Gy radiation had a significantly better outcome (pooled HR = 0.75, 95% CI: 0.63–0.91, *P* = 0.003, Figure 4B), despite the significant heterogeneity inside this subgroup (*P* < 0.000001, I^2^ = 85%). These results suggest that ≥ 60 Gy radiation might be an optimal dose for Asian EC patients.

### Association of radiation dose with treatment failure

**Figure 5:**
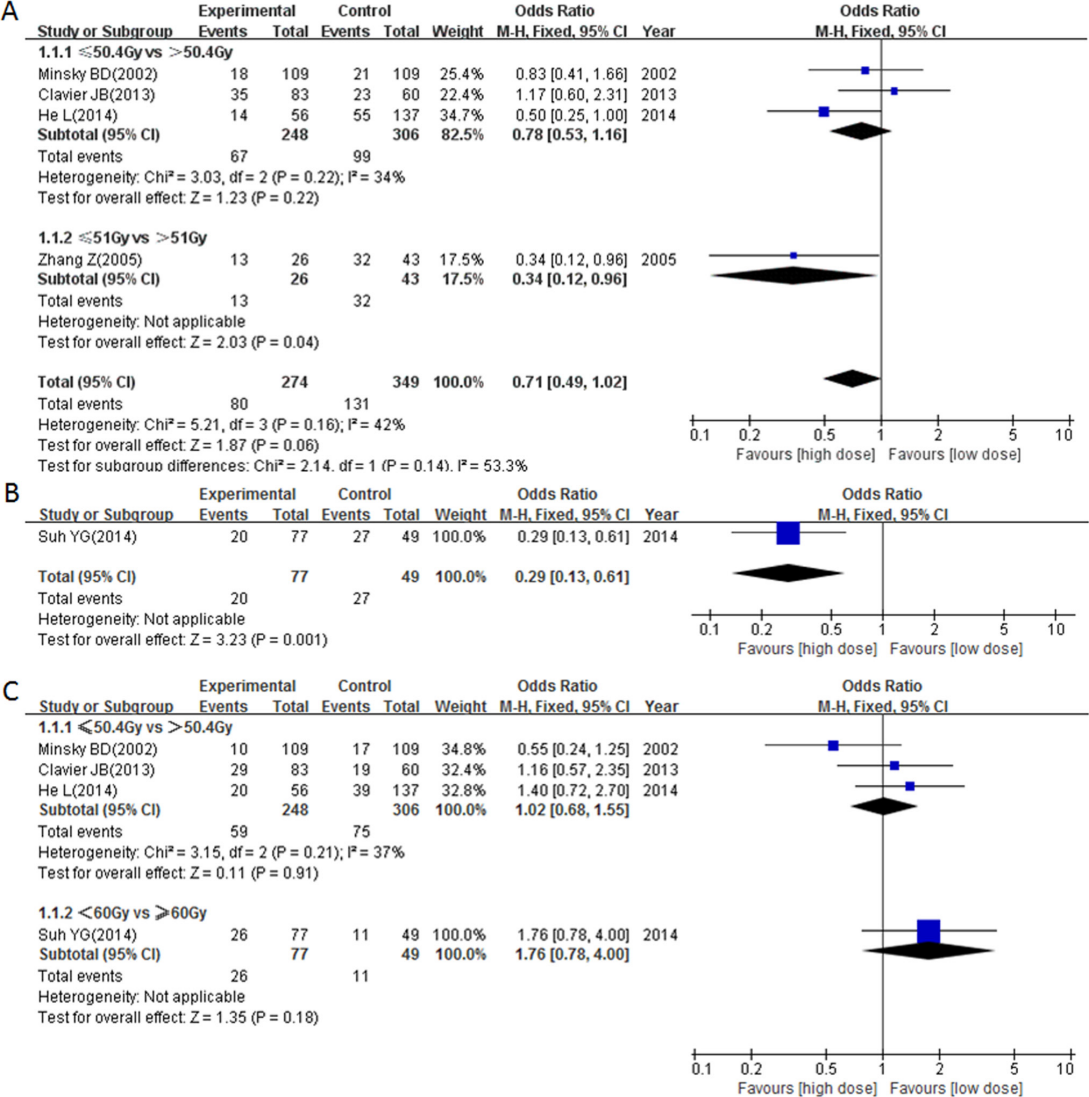
Forest plot describing the association between locoregional control and (< circa 50 Gy vs ≥ circa 50 Gy) subgroup (**A**) and (< 60 Gy vs ≥ 60 Gy) subgroup (**B**) and the association between distant metastasis control and different radiation doses (**C**).

A total of 5 studies presented data for the exact frequency of LRF. There were 4 studies comparing odds ratios (ORs) between radiation dose > 50.4/51 Gy with ≤ 50.4/51 Gy, and the results showed that the HD group (> 50.4/51 Gy) had a nearly significant association with higher locoregional control (pooled OR = 0.71, 95% CI = 0.49–1.02, *P* = 0.06, Figure 5A). More so, patients that received ≥ 60 Gy radiation had higher locoregional control (OR = 0.29, 95% CI: 0.13–0.61, *P* = 0.001, Figure 5B).

A total of 4 studies reported data for exact frequency of DMF. Our results showed that neither the > 50.4 Gy group nor the ≥ 60 Gy group was associated with good distant metastasis control (pooled OR = 1.02, 95% CI: 0.68–1.55, *P* = 0.91 and OR = 1.76, 95% CI: 0.78–4.00, *P* = 0.18 respectively, Figure 5C).

### Publication bias

**Figure 6:**
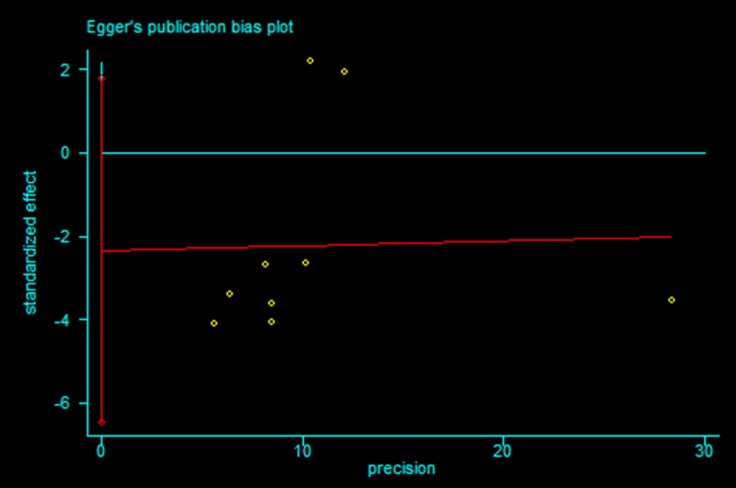
Egger’s publication bias plot for (< 60 Gy vs ≥ 60 Gy group) meta-analysis

**Figure 7:**
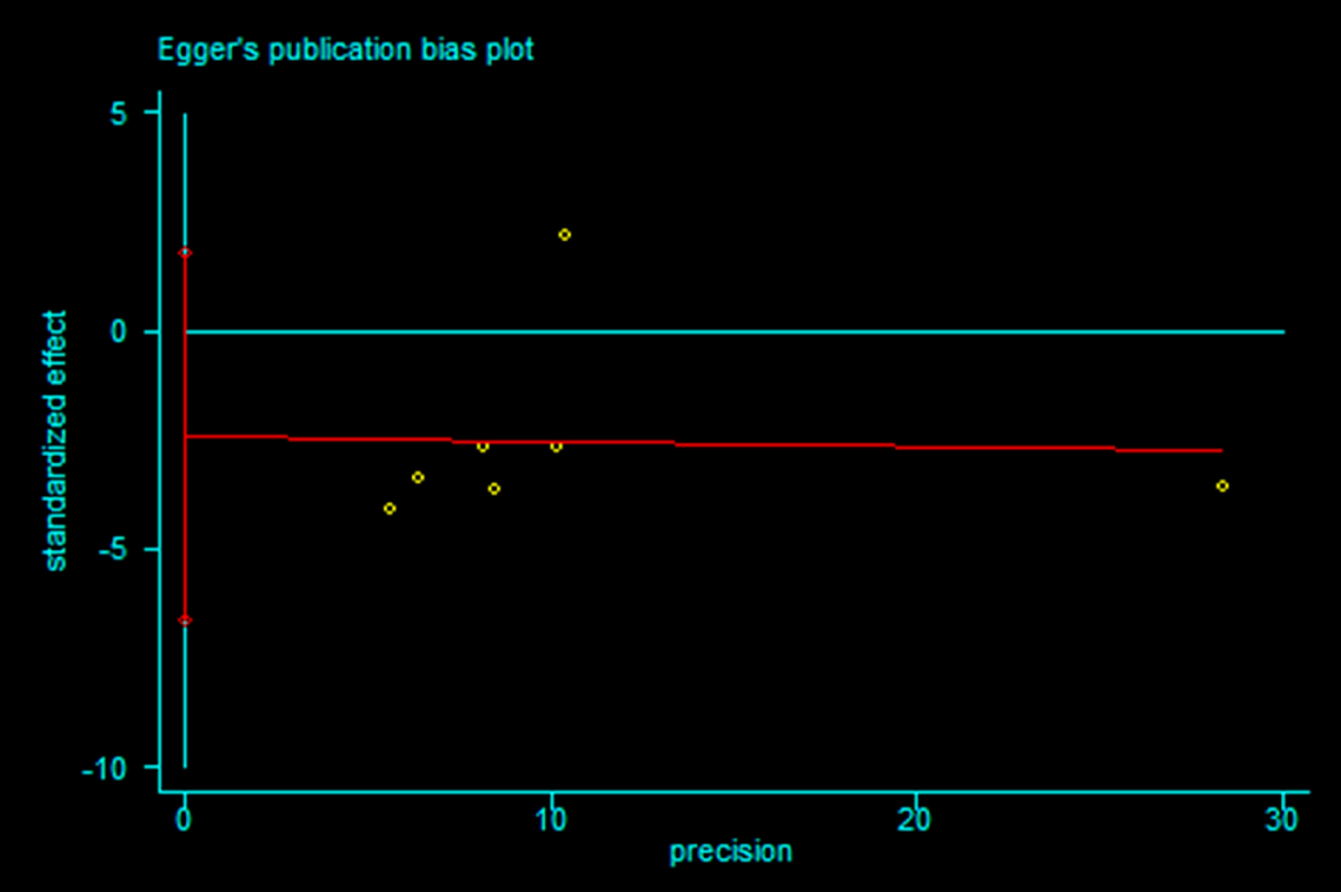
Egger’s publication bias plot for (< 60 Gy vs ≥ 60 Gy subgroup) meta-analysis from Asian countries

Publication bias statistical analysis was performed using the Egger’s test. No publication bias was detected in meta-analysis of < 60 Gy vs ≥ 60 Gy group and < 60 Gy vs ≥ 60 Gy subgroup from Asian countries (*P* = 0.220 and 0.200 respectively, Figures 6 and 7).

## DISCUSSION

Definitive CCRT is considered as the optimal choice and as the standard of care in non-operable EC patients. Interestingly, the recommended dose of RT still remains controversial. In this meta-analysis, we investigated the correlation between clinical benefits and radiation dose in non-operable EC cases. The results of the current analysis suggest that a higher radiation dose could bring about better locoregional control as compared to lower doses. For curative purpose, the optimal radiation dose should not be lower than 50 Gy and a total dose of ≥ 60 Gy could improve patients’ OS, especially those in Asia. An extremely high radiation dose of 70 Gy did not result in extra benefit or clinical outcome.

Thus far, the INT-0123 trial in USA was the only RCT that compared different radiation doses in combination with chemotherapy for non-surgical EC patients [4]. In that trial, results showed that a 14.4 Gy dose escalation did not result in either OS benefit or locoregional control benefit. Although 11 treatment-related deaths happened in the HD arm compared with 2 in the standard dose arm, the authors of INT-0123 indicated that the treatment-related deaths did not seem to be related to the higher radiation dose. Additionally, the HD arm of the study had a significant prolongation of treatment time because of toxicity breaks as well as a significantly lower actual dose of 5-Fluorouracil (5-FU) than the LD arm, which might have contributed, in part, to the lack of benefit for patients who received HD treatment. Because of this highly controversial result and the absence of similar RCT, CCRT with 50.4 Gy for locally advanced EC has been considered as standard treatment for almost 14 years in most Western countries. However, with the clinical application of more precise radiotherapy techniques such as three-dimensional-conformal or intensity-modulated radiotherapy, the issue of additional dose escalation seems to be of potential value. Many of the studies that investigated the treatment of EC patients successfully administered a total dose of > 50 Gy with decreased treatment-related toxicity [26, 27], no excess morbidity [28] or well tolerated toxicity with concurrent chemotherapy [29–32], even in elderly patients (70∼87 years old) [33]. Still, many prospective and retrospective studies have evaluated a CCRT dose ≥ 60 Gy in clinical practice. A recent systematic review showed that there at least 27 studies investigated the clinical efficiency of ≥ 60 Gy combined with cisplatin-based concurrent chemoradiotheray from 1990 to 2014 [8]. From this review, it was worthy to mention that a large number of these studies indicated that a higher radiation dose brought about a clinical benefit without a significant increase in radiation related toxicities, which is contradictory to INT-0123 trial. However, it was also important to state that this review was a compilation of single arm series or single institution, which meant that only 3 included studies contained both a HD (≥ 60 Gy) arm and a conventional-dose (50–54 Gy) arm, while other studies contained only one of these treatment arms. Therefore, the potential patient selection biases, publication bias and heterogeneities in radiation techniques, tumor response evaluation criteria, the state of life evaluation criteria and study periods might have influenced the pooled results and made the interpretations challenging. In the present study, we had attempted to collect studies which contained both HD and LD arms to reduce bias and heterogeneity, and performed the meta-analysis to find any evidence of treatment benefit in HD arm. In addition, most HRs with their 95% CIs for OS in the HD arm originated from multivariate Cox proportional-hazards regression analysis, which indicates that the two groups could be considered comparable on each variable used in the adjustment. Consequently, with this systematic review [8], we hope that this study opens up discussions amongst radiologists about the need to still explore optimal radiation doses in clinical practice.

Theoretically, a radiation dose of 60 Gy or higher is needed to abrogate a tumor mass [8, 34]. Our results showed that 50–70 Gy seemed to be a rational curative dose for EC patients based on a limited number of studies. Early in 1989, Sun [35] made a 10-year follow-up survey on 869 patients with esophageal carcinoma who received ^60^Co radical radiation, and found that a total dose of ≥ 60 Gy brought about better survival rate when compared with patients receiving 50–59 Gy radiation. In 2015, Song and colleagues [8] conducted a systematic review in a large group of patients worldwide and found that ≥ 60 Gy CCRT (cisplatin-based) improved clinical outcomes (response rate, local regional recurrence rate distant failure rate and overall survival) relative to the conventional dose (50∼54 Gy) group, especially for esophageal squamous cell carcinoma. In our analysis however, we didn’t find a benefit of ≥ 60 Gy CCRT relative to 50∼50.4 Gy based on only 3 studies. In addition to the prolonged treatment time of HD arm in the INT-0123 trial, another possible explanation might be that there was a significantly higher rate of salvage surgery after progression in 50.4 Gy CCRT group compared with the ≥ 60 Gy group in Hirano’s retrospective study [25], which might have also led to a prolongation in OS in the 50.4 Gy group. However, when more studies were included (< 60 Gy vs ≥ 60 Gy), a significant OS benefit for those treated ≥ 60 Gy was found as compared to those with < 60 Gy. As with Song’s study [8], different geographic areas used different radiation doses. For instance, a radiation dose ≥ 60 Gy was more widely used in Asian countries as compared to Western countries. From the subgroup analysis according to geographic area, we found that ≥ 60 Gy radiation would be an optimal dose for Asian EC patients, while the optimal radiation dose for Western EC patients is still unclear. With the utilization of more advanced radiation technology platforms (e.g. three-dimensional planning radiation), a survival benefit may yet be seen for Western EC patients. Certainly, our results reflect the rationale behind the three currently ongoing clinical trials evaluating HD and LD treatment options; NCT01348217: 50 Gy vs 66 Gy (since 2011, France); NCT01937208: 50 Gy vs 60 Gy (since 2013, China) and NCT02556762: 50 Gy vs 66 Gy (since 2015, China). Additional information is available on the clinical trials registry website, www.clinicaltrials.gov.

Locoregional failure is the major form of treatment failure in EC, occurring more often in tumors of large volume [36–38]. Welsh J et al., [36] reported that unresectable EC with gross tumor volume (GTV) failure was associated with a shorter survival when compared to patients without GTV failure, suggesting that local control was very important for the improvement of survival. According to a previous study [19], a radiation dose of 50.4 Gy or less is insufficient to obtain a satisfactory local control for definitive CCRT purpose if surgery is not done. In contrast to INT-0123 trial, a higher radiation dose was significantly associated with a better local/ locoregional control [8, 12, 39]. In our present meta-analysis from limited studies, we have found that high radiation dose was associated with better locoregional control but not distant metastasis. Before the results of the ongoing RCT become available, we believe our results could provide some potential practical suggestions for EC patients worldwide.

Importantly, there are several limitations to our study. Firstly, only 18 studies were included, most of which were retrospective studies except one RCT and one population based propensity-score matched analysis. Secondly, many studies had a relative small amount of patients in each group. Third, there were significant heterogeneities in many subgroup meta-analysis. Forth, the power of Egger’s test for publication bias is underpowered if the number of trials is less than 10. These factors might have influenced our findings and as a consequence, our inferences. Indeed, more RCTs are needed to further support our conclusions.

Taken together, our results suggest that a higher radiation dose could bring about better locoregional control than LD therapy. For curative purpose, the rational radiation dose appears to be 50–70 Gy and a total dose of ≥ 60 Gy could improve OS, especially in Asian patients. This meta-analysis supports a radiation dose of ≥ 60 Gy in the treatment of EC. In the future, further insights into the long-term benefits and clinical significance of high dose radiation will be provided by the three ongoing RCTs mentioned above.

## MATERIALS AND METHODS

### Literature search, identification and selection

All studies published before November, 2016 that investigated the association of radiation dose and curative efficiency in EC were considered in this meta-analysis. A comprehensive literature search was carried out in PubMed and Embase. The search terms used were: (“esophageal”[Title]) or (“oesophageal”[Title]) or (“esophagus”[Title])) and (“tumor”[Title]) or (“cancer”[Title]) or (“carcinoma”[Title]) or (“neoplasm”[Title]) or (“neoplasms”[Title])) and (“radiotherapy”[Title]) or (“radiation”[Title]) or (“chemoradiation”[Title]) or (“chemoradiotherapy”[Title]) or (“radiochemotherapy” [Title]) or (“irradiation” [Title]) or (“chemo-irradiation”[Title]) or (“chemo-radiotherapy”[Title])) and (“dose” [Abstract]). There was no language restriction.

### Endpoints of interest

The primary endpoints considered were frequency of LRF and DMF, HRs with their 95% CIs for OS after treatment. Patients were classified by radiation dose using a threshold as defined by the individual studies.

### Data extraction and synthesis

Data were extracted from the primary publications. Two authors (Y Chen and HP Zhu) independently reviewed the studies to exclude irrelevant or overlapping studies and extracted the data from all included studies. For each study, the following details were extracted: name of the first author, year of publication, sample size, age of the patients, study period, geographic area, radiation technology, radiation dose of whole group, chemotherapy regimens, radiation dose in LD and HD groups, pathological types, clinical stage and median follow-up time. Disparities were discussed until a consensus was reached. The frequencies of LRF and DMF from the different groups were expressed as an OR with its 95% CI. If a figure for HR and 95% CI was not available, an estimate value was calculated indirectly by using the methods described by Tierney et al. [40]. Survival rates from Kaplan-Meier curves were read using Engauge Digitizer version 4.1 (available from: http://digitizer.sourceforge.net/) and the resulting data were then entered in the calculation spreadsheet appended to Tierney’s paper.

### Quality assessment

For non-randomized studies, the 9-star Newcastle-Ottawa Scale (Available from: http://www.ohri.ca/programs/clinical_epidemiology/oxford.htm) was performed to assess each study. The quality categories were defined as follows: high quality (score 7–9), medium quality (score 4–6) and low quality (score less than 4) [41]. Quality of RCT was assessed by the 7-point JADAD scale [42]. This scale contains the following categories: randomization, concealment of allocation, double blinding, withdrawals, and dropouts. Total scores 1 to 3 mean low quality and 4 to 7 mean high quality [43].

### Statistical analysis

RevMan 5.3 analysis software (Cochrane Collaboration, Copenhagen, Denmark) was used to obtain pooled statistics for HRs of OS or ORs of LRF and DMF. The statistical significance of the pooled estimates was examined by a *Z* test. Statistical heterogeneity was assessed using the Cochran‘s Q and I^2^ statistics as concluded by Chen et al [44]. Briefly, the percentages of I^2^ = 25%–50%, 50%–75%, and > 75% mean low, moderate and high heterogeneity, respectively with I^2^>50% suggesting significant heterogeneity. A random-effect model was conducted to analyze the estimates when significant heterogeneity was found among the studies; otherwise, a fixed-effect model was used. Publication biases among the studies were performed. Egger’s publication bias plots of (< 60 Gy vs ≥ 60 Gy) meta-analysis and (< 60 Gy vs ≥ 60 Gy from Asian countries) subgroup meta-analysis were used to find any evidence of publication bias. Egger’s test, estimated by STATA (version 12.0; StataCorp, College Station, Texas, USA), was performed to measure the publication bias. Statistical significance was defined as *p* < 0.05. Subgroup analyses were performed according to threshold of radiation dose and the different geographic areas.
